# Strategies for mitigating emerging artemisinin-based antimalarial drug resistance in Rwanda: a promising approach for managing therapies in malaria-endemic countries

**DOI:** 10.1136/bmjgh-2025-020884

**Published:** 2025-10-02

**Authors:** Claude Mambo Muvunyi, Pierre Gashema, Aimable Mbituyumuremyi, Patrick Gad IRADUKUNDA, Emmanuel Edwar Siddig, Jean Damascene Niyonzima, Emmanuel Hakizimana, Noella Umulisa, Abdisalan Mohamed Noor, Jules Mugabo Semahore, Albert Tuyishime, Jean de Dieu HARELIMANA, Jeanne Umuhire, Yvan Butera, Sabin Nsanzimana

**Affiliations:** 1 Rwanda Biomedical Center, Kigali, Rwanda; 2 Africa Centres for Disease Control and Prevention, Addis Ababa, Ethiopia; 3 Repolicy Research Centre, Kigali, Rwanda; 4 Quality Management Division, Rwanda FDA, Kigali, Kigali City, Rwanda; 5 Jhpiego Kigali, Rwanda, Kigali, Rwanda; 6 Harvard University, Cambridge, Massachusetts, USA; 7 World Health Organization Kigali, Rwanda, Kigali, Rwanda; 8 Republic of Rwanda Ministry of Health, Kigali, Kigali City, Rwanda

**Keywords:** Health policy, Parasitology, Treatment, Malaria

## Abstract

Malaria treatment failures associated with reduced efficacy of chloroquine (CQ) and amodiaquine (AQ) antimalarial drugs emerged in Rwanda during the 1980s, prompting the policy shift towards adopting artemisinin-based combination therapies in 2006 as an alternative. However, recent findings from malaria surveillance and therapeutic efficacy studies have revealed a countrywide increase in antimalarial drug resistance. Particularly, artemether-lumefantrine (AL) efficacy has significantly decreased, probably due to the emergence of *Plasmodium falciparum* (*Pf*) genomic mutations. To mitigate the current drug resistance, Rwanda has adopted targeted multiple first-line therapies. Through the national malaria control program, antimalarial drugs were deployed in accordance with the reported resistance profile. A significant rise in *Pfkelch13* mutations, particularly A675V associated with AL resistance, was mainly reported in the western region; therefore, artesunate-pyronaridine was recommended. Dihydroartemisinin-piperaquine was considered in eastern and central regions, where *R561H* mutations were predominant. On the contrary, AL was maintained in the southern region, where the prevalence of the *R561H* mutation was low. Insights from this data-driven model will inform its extension to other malaria-endemic countries facing emerging *Pf* genetic diversity.

**Summary box**

- In 2006, Rwanda adopted artemisinin-based combination therapies (ACTs) for malaria treatment in response to increasing treatment failures and growing resistance to chloroquine and amodiaquine.
- Emerging *Pfkelch13* mutations are compromising ACT efficacy, with surveillance data indicating decreased artemether-lumefantrine therapeutic response.
- A data-driven, region-specific multiple first-line therapy (MFT) approach has been implemented to mitigate antimalarial resistance and sustain treatment efficacy.
- Rwanda’s adaptive MFT model provides a potentially scalable framework for managing malarial drug resistance in endemic settings.

## Introduction

 Malaria continues to pose a significant public health challenge in Africa, accounting for about 95% of global cases and 96% of malaria-related deaths, primarily among children under 5 years old.^1^ Despite substantial progress in malaria control over the past two decades largely due to the widespread use of artemisinin-based combination therapies (ACTs), insecticide-treated bed nets and improved diagnostic tools, emerging drug resistance now threatens to reverse these gains.^2^ ACTs have been the first-line treatment for uncomplicated *Plasmodium falciparum* (*Pf*) malaria since the early 2000s.^3^ Rwanda Ministry of Health adopted ACTs in national treatment guidelines in 2006.^4^ However, resistance to artemisinin and its partner drugs has been increasingly reported in the East African region, including Rwanda.^5^ Artemisinin resistance was reported in Rwanda from the sample collected in the therapeutic efficacy study (TES) in 2014, when delayed parasite clearance was observed in some patients treated with AL.^6^ Further molecular surveillance confirmed the presence of *Pfkelch13* mutations, particularly R561H, A675V and P574L associated with partial artemisinin resistance countrywide.^7^,^10^ Studies in Rwanda have since reported treatment failure rates ranging from 5%–10% in the central region of Rwanda (Kicukiro), eastern region (Kayonza) and western region (Rusizi). These findings provided early warnings of reduction efficacy in artemether-lumefantrine (AL).^6^ To proactively address this growing threat of antimalarial resistance, Rwanda, through the national malaria control programme (NMCP), introduced the targeted multiple first-line therapies (MFTs). As previously reported, adoption of a diversified ACT approach may maintain treatment efficacy.^11^ The MFT approach includes dihydroartemisinin-piperaquine (DHA-PPQ) as a promising alternative due to its longer half-life, which provides extended post-treatment prophylaxis, reducing reinfection rates.^12^ Artesunate-pyronaridine (AS-PY) has demonstrated high efficacy in clinical trials and is particularly valuable in areas where AL efficacy is compromised.^13^ These strategies aimed to reduce selective pressure on any single ACT, slow resistance development and sustain treatment efficacy.

## Historical perspectives on malaria treatment and emergence of drug resistance in Rwanda

Rwanda’s antimalarial treatment landscape has evolved over decades. In the 1980s, CQ and AQ failure rates escalated, leading to policy shifts supported by the East African Network for Monitoring Antimalarial Treatment (EANMAT).^14^,^16^ With initial support from EANMAT, and subsequently by other partners such as the WHO, Global Fund and the US President’s Malaria Initiative (PMI), the NMCP has been closely monitoring the efficacy of antimalarial drugs since 2002. This evidence has informed periodic changes in treatment guidelines. Table 1 summarises studies conducted in Rwanda between 2002 and 2022 that evaluated the efficacy of antimalarial treatments. The majority of the studies (9 out of 14) were open-label randomised clinical trials. Additional study types included two TES, one comparative clinical trial, one multicentre comparative efficacy study and one individual patient data (IPD) meta-analysis of randomised controlled trials (RCTs). Four main treatment regimens were assessed: AL, amodiaquine plus artesunate (AQ+AS), DHA-PPQ and amodiaquine plus sulfadoxine-pyrimethamine (AQ+SP). Efficacy outcomes were primarily reported at day 28 for AL, AQ+SP and AQ+AS and at day 42 for DHA-PPQ. In 1999, efficacy studies showed that the clinical failure of CQ and SP in the country exceeded the WHO cut-off threshold of below 90%, necessitating a change in drug policy.^17^ In response, the Rwandan Ministry of Health (MoH) revised its treatment guidelines in 2001 to favour AQ combined with SP to uphold treatment efficacy. However, by 2000, studies showed that the efficacy of AQ and AQ+SP dropped below acceptable levels, with treatment failures exceeding 20%.^18 19^ After 6 years of implementation and with the increase of resistance to AQ+SP, the MoH shifted to AL after studies showing high efficacy in 2006.^4^ Between 2003 and 2009, high efficacies of the other combinations of artemisinin compounds with either SP, AQ or piperaquine were also reported, above 95% in most of the study sites.^20^,^25^ In the period of 2012 and 2019, different studies conducted in several sites revealed no evidence of delayed clearance and treatment failure rates above 10% for AL or DHA-PPQ.^6 10^ However, TES conducted in 2022 revealed 16% of treatment failure for AL in a site in Ngoma in the Nyaruguru district, Southern region.^26^ While this suggests a potential decline in clinical efficacy, it is important to note that. Since lumefantrine has never been presented as a monotherapy and a stable, well-defined lumefantrine-resistant phenotype has not yet been identified, AL has remained comparatively successful since the start of its use.^27^ Despite its effectiveness, studies in Rwanda have shown the emergence and spread of *Pfkelch13* mutations, particularly R561H, A675V and C469F, across the country.^8^ The decreasing susceptibility of *Pf* to lumefantrine has been associated with mutations in the *pfmdr1* gene, particularly haplotypes involving the N86 and D1246 alleles as well as the wild-type K76 allele of the *pfcrt* gene; notably, these genetic markers previously linked to reduced lumefantrine efficacy have been reported in Rwanda and Uganda.^28 29^ A longitudinal study in Rwanda revealed high frequencies of A675V at 35.5% in the western region (Mushubati in Rutsiro district) and 27.2% in western region (Byahi in Rubavu District) nearby eastern DRC.^8^ R561H was also reported on average at 19% in 20 health facilities, with Kibingo health centre in Nyamasheke district western region at 54.5% and other areas such as Tanda health centre in Gicumbi district in Northern region at 33% and Bugaragara health centre in Nyagatare District in eastern region at 50%.^8^ Additionally, Malaria Molecular Surveillance study conducted using 2014–2015 Demographic Health Survey samples revealed R561H in eastern region (Kirehe and Ngoma districts) at 5.5% and 2.74%, respectively.^9^ 22% for R561H was also reported in two sites in central region in Kigali.^30^ R561H mutation was also confirmed at 17% in central region (Masaka in Kigali) and 19% in eastern region (Rukara in Kayonza).^6^ In southern region (Huye district), R561H was reported at 12.1% in 2021.^31^ The trends observed in Rwanda have also been reported in neighbouring countries. For instance, Uganda has reported evidence of K13 mutations ranging from 4.5% in 2016 to 25.5% in 2022. Dominant mutations in northern Uganda were C469Y and A675V, while P441L, C469F and R561H were most common in the western and southwestern regions.^32^ In northwest Akagera, Tanzania, the K13 R561H mutation was detected at 7.7%, with parasite isolates genetically linked to Rwanda 2015 strains, indicating potential regional spread.^33^ In light of this regional expansion of resistance-associated mutations and the documented AL treatment failures, there is an urgent need to introduce alternative ACTs to safeguard malaria treatment efficacy across Rwanda and the wider region.

**Table 1:** Summary of antimalarial therapeutic efficacy studies in Rwanda, 2002–2022

Drug tested	Study design /blinding	Target population	Year and reference	Health facilities (region)					
				Rukara (eastern) % (n cases)	Masaka (Kigali) % (n cases)	Bugarama (western) % (n cases)	Nyarurema/ (eastern) % (n cases)	Ruhuha/ (eastern) % (n cases)	Ngoma/ (southern) % (n cases)
AQ*	Comparative clinical trial	6–59 months	2002^18^	72% (32/44)	72% (31/43)	NA	NA	NA	NA
AS+AQ*	RCT	6–59 months	2002^19^	80% (37/46)	96% (45/47)	NA	NA	NA	NA
DHA-PPQ†	RCT/open label	12–59 months	2003–2 00^20^	89% (78/88)	99% (74/75)	99% (86/87)	NA	NA	NA
AL*	RCT/open label	12–59 months	2004^4^	94% (92/98)	NA	99% (141/143)	NA	NA	NA
AL*	RCT/open label	Above 6 months	2006^41^	NA	100% (164/164)	NA	NA	NA	NA
AL*	RCT/non-inferiority	6–59 months	2007–2 00^42^	90% (64/71)	NA	99% (74/75)	NA	NA	NA
DHA-PPQ†	RCT/non-inferiority	6–59 months	2007–2 00^42^	97% (68/70)	NA	95% (72/76)	NA	NA	NA
AS+AQ*	Multicentre, comparative efficacy studies	75% under 5 years	2008^22^	88% (121/138)	95% (116/122)	95% (142/150)	NA	NA	NA
DHA-PPQ†	Individual patient data (IPD) meta-analysis of RCTs	Under 5 years	2008^21^	88% (76/86)	97% (82/84)	97% (80/82)	NA	NA	NA
AL*	RCT/open label	6–59 months	2015^43^	99% (123/124)	97% (107/111)	96% (97/101)	97% (61/63)	98% (86/88)	NA
DHA-PPQ†	RCT/open label	6–59 months	2015^43^	99% (129/128)	97% (117/121)	NA	NA	NA	NA
AL*	Therapeutic efficacy study	6–59 months	2018^6^	94% (65/69)	97% (48/50)	97% (74/76)	NA	NA	NA
AL*	Phase 2 RCT	Not reported	2019^30^	NA	94% (16/17)	NA	NA	NA	NA
AL*	Therapeutic efficacy study	6–59 months	2022^26^	NA	84.1% (74/88)	93.2% (82/88)	NA	NA	96.6% (85/88)

PCR correction was used to distinguish recrudescence from new infections, in line with the WHO protocols.

* Efficacies are reported at day 28;

† Efficacies are at day 42 due to the longer terminal half-life of piperaquine.

AL, artemether-lumefantrine; AQ, amodiaquine; AS, artesunate; DHA, dihydroartemisinin; PPQ, piperaquine; RCT, randomised controlled trial.

## Data-driven approach for managing artemisinin-based drug resistance in Rwanda

**Figure 1:**
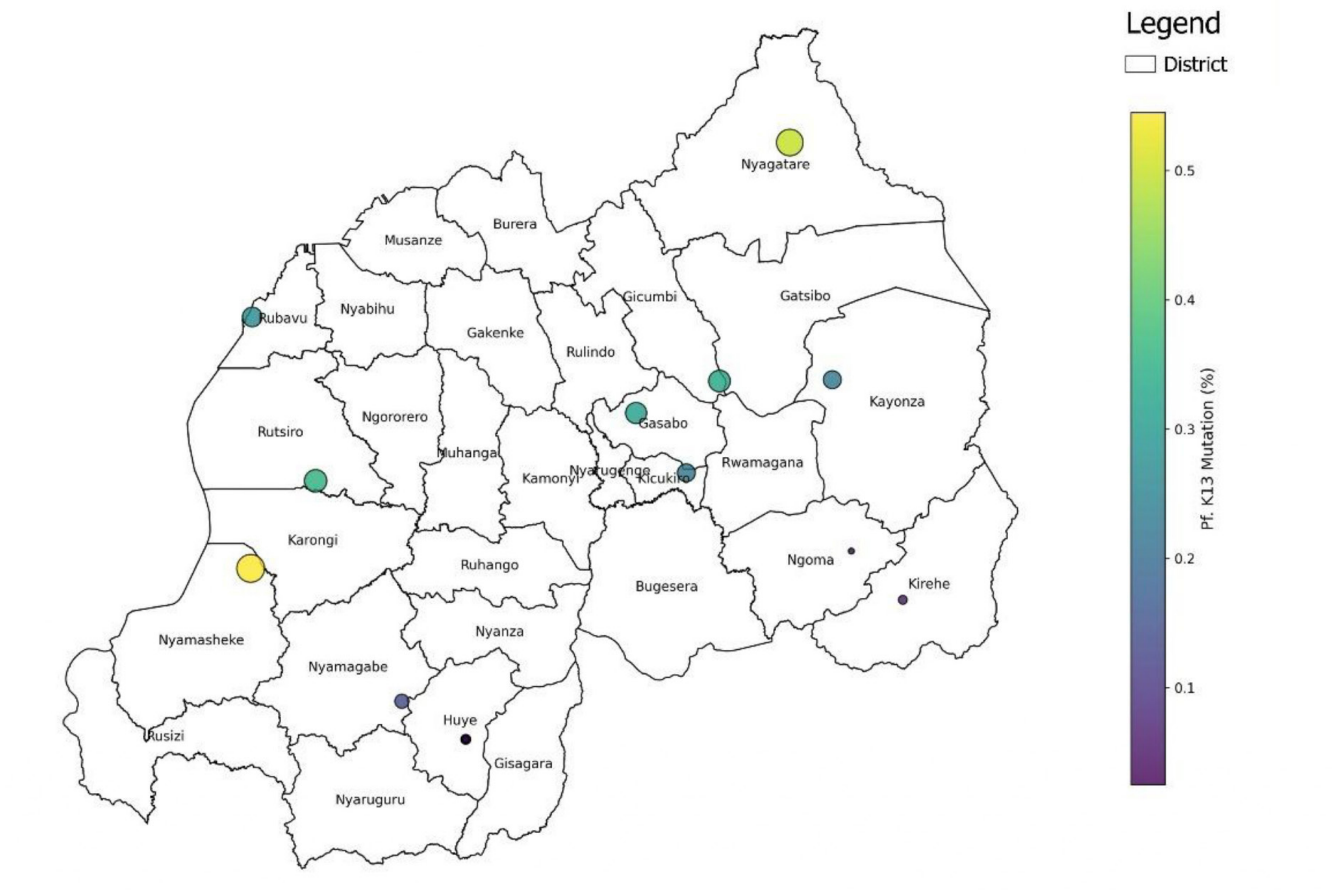
Distribution of *Plasmodium falciparum.* 13 mutations in Rwanda (2014–2024). The size of each point and intensity of its colour correlate with the frequency of mutations.

Rwanda's transition from AL to MFT aligns with the WHO’s implementation framework for MFT^11^ and is grounded in a comprehensive review of malaria treatment efficacy and policy responses from 1980 to 2024. Key data sources included TES, molecular surveillance reports of Pfkelch13 mutations, national malaria strategic plans and WHO policy guidance. Policy changes were mapped against declining antimalarial efficacy (table 1**:** summary of antimalarial therapeutic efficacy studies in Rwanda, 2002–2022.) The geographic distribution of resistance markers, particularly R561H, A675V and C469F, is also illustrated. K13 mutations were most prevalent in Nyagatare (eastern region) and Nyamasheke (western region) (figure 1).

**Figure 2:**
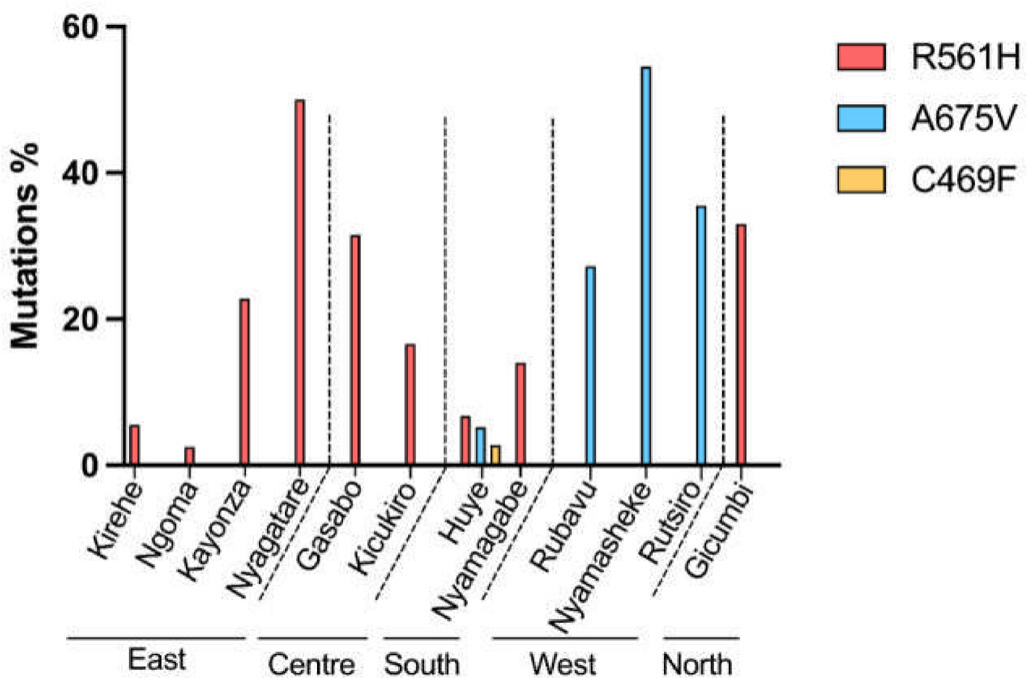
Frequency of *Plasmodium falciparum* mutations in various regions of Rwanda.

The frequency of mutations ranged from 2.5%–50% in the eastern region, 16.6%–31.5% in the central region, 2.8%–14% in the southern region, 27.2%–54.5% in the western region and approximately 33% in the northern region (figure 2). Evidence from TES and molecular surveillance informed multistakeholder consultations with the MoH, the WHO Rwanda Office, the PMI, the Global Fund and the National Malaria Technical Working Group. Recommendations from the Rwanda Malaria Mid-Term Review^34^ and annual malaria and neglected tropical diseases reports supported the strategic shift to MFT.^35^

Subsequent adoption, a cascade training model was deployed to capacitate healthcare workers and community health workers, who transitioned from binome to polyvalent roles. Community engagement was facilitated through citizens’ assemblies and monthly Umuganda activities to promote awareness and treatment adherence.

**Figure 3:**
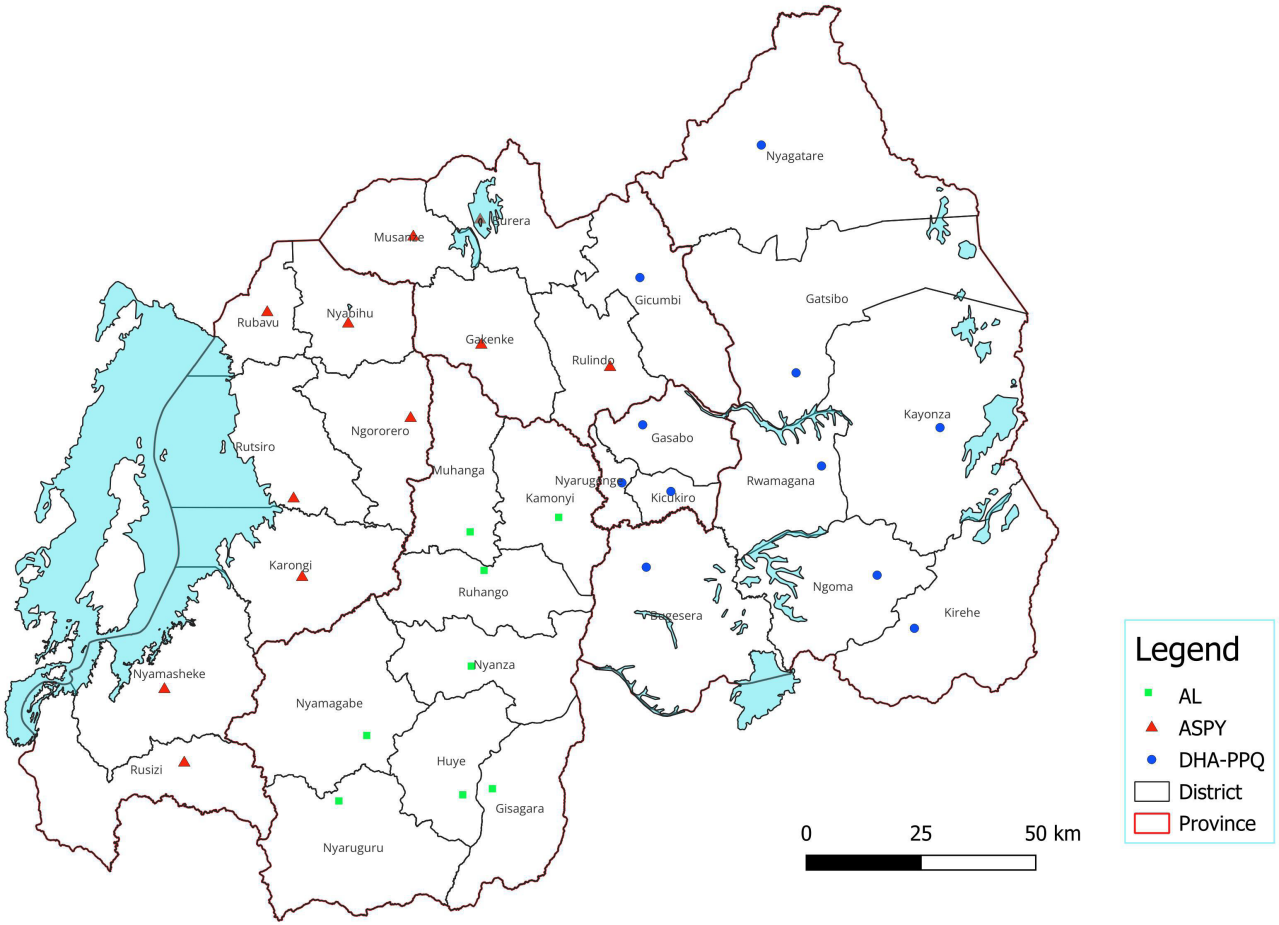
Deployment plan of multifirst-line therapy in Rwanda March 2025. Blue point: dihydroartemisinin-piperaquine (DHA-PPQ), red triangle: artesunate-pyronaridine (AS-PY), green square: artemether-lumefantrine (AL).

To support implementation, the NMCP revised procurement and distribution systems to prevent stockouts and expiries. Drone-enabled supply chains, in partnership with Zipline, ensured timely delivery of antimalarials to remote areas.^36^ Deployment of AL, AS-PY and DHA-PPQ was stratified by regional resistance patterns: DHA-PPQ was introduced in high R561H prevalence districts in the Eastern and Kigali City regions, covering 44.7% of the population; AS-PY was prioritised in the Western region, particularly Rubavu, Rutsiro and Nyamasheke, where A675V was dominant (26.4% coverage), and AL was retained in Southern districts with low resistance markers, covering the remaining 29% (figure 3: MFT deployment plan). All analyses were performed using the GraphPad software V.10.4.1 (2024) and Python V.3.13.3 (2025).

## Challenges and considerations

The transition to an MFT approach represents a proactive measure against emerging drug resistance, yet several significant challenges must be surmounted to ensure its effective implementation and long-term success. One of the foremost challenges pertains to drug availability and supply chain management. Ensuring consistent access to all three ACTs—AL, AS-PY and DHA-PPQ—across various health facilities is critical. This necessitates the establishment of robust procurement and distribution systems capable of managing a diversified drug portfolio efficiently. To address these logistical concerns, the NMCP is leveraging advanced technology and innovative strategies, as outlined in the WHO MFT implementation guide.^11^ Rwanda is leveraging the existing supply chain system based at the district pharmacy to optimise on-time distribution of malaria treatment and additionally drone-based technology, which is being used for timely delivery of essential antimalarial drugs to hard-to-reach areas as supplementary strategies. This combined strategy will prevent stockouts and ensure uninterrupted access to effective treatments.^36^ In addition, this approach will not only enhance efficiency but also minimises delays that could exacerbate the burden of malaria in affected communities.^11^ Sustainability of malaria treatment efficacy will require long-term investments in surveillance of antimalarial drug efficacy, strong regulatory system on the use of diagnostic and therapeutic critical interventions to limit the spread of drug parasite resistance and research innovation with a focus on existing tools and the development of new tools to help in the monitoring of emerging resistant strains, which are outlined as key pillars for responding to antimalarial drug resistance in Africa.^37 38^ In line with this, Rwanda envisions a robust framework for long-term surveillance of antimalarial drug efficacy as critical to sustaining malaria control achievements and guiding evidence-based policy decisions. The NMCP, with support from the Bill & Melinda Gates Foundation beginning in January next year, will implement an integrated surveillance strategy that includes routine therapeutic efficacy monitoring of ACTs through standardised in vivo studies; mapping the prevalence and geographic distribution of validated and candidate resistance markers such as *Pfkelch13* mutations (eg, R561H, A675V); tracking partner drug resistance markers like *plasmepsin 2/3* amplifications; analysing treatment failure rates stratified by region and age using DHIS2 data and assessing trends in malaria incidence and case fatality to triangulate with resistance data. The strategy also includes strengthening national laboratory capacity and introducing advanced molecular technologies to distinguish recrudescence from new infections, monitor both drug and insecticide resistance, detect *Pfhrp2/3* deletions and conduct ex vivo drug sensitivity assays. These indicators will serve as critical benchmarks for evaluating the impact of implemented malaria control strategies over time. On the other hand, affordability remains another concern, necessitating a well-comprehensive cost-effectiveness analysis to optimise resource allocation. In this context, the MoH and NMCP are working with Jhpiego Rwanda on the STOP-AMDR initiative (Scaling the Optimal Use of Multiple ACTs to Prevent Antimalarial Drug Resistance), which supports the MFT strategic implementation.

Moreover, integration with broader malaria elimination strategies is essential; treatment diversification must be complemented by enhanced vector control, rapid diagnostic testing and cross-border collaboration to prevent the spread of resistant strains. Countries such as Cambodia and Thailand, which have implemented MFT policies, have reported a 30%–50% reduction in treatment failure rates, underscoring the potential impact of this approach if properly executed.^39 40^

Community engagement is another essential consideration. Although public awareness campaigns and initiatives like citizens' assemblies meeting through local government can educate the community about new treatment options and adherence to guidelines, achieving substantial changes in behaviours and attitudes towards malaria treatment requires sustained community involvement. Ensuring that healthcare providers are well-trained and equipped to implement the MFT strategy is vital to disseminating accurate information and fostering trust within communities.

## Conclusion

In summary, addressing the emerging threat of artemisinin resistance in Rwanda demands a multifaceted, adaptive and well-coordinated approach. The implementation of targeted MFT tailored according to regional resistance profiles represents an innovative and promising strategy that can prolong the efficacy of antimalarial treatments. Rwanda’s experiences demonstrate that integrating molecular surveillance, strategic drug deployment, community engagement and advanced logistics (such as drone delivery) fosters resilience against resistance development. However, sustaining these gains requires continuous investment in robust surveillance systems to detect resistance patterns early, strengthening regulatory frameworks to ensure rational drug use and expanding regional collaboration to prevent cross-border spread of resistant strains. Equally vital is community participation and health worker training to ensure adherence to treatment guidelines.

Moving forward, insights from Rwanda’s strategic model will inform its adaptation and implementation in other malaria-endemic countries facing similar challenges. Success hinges on sustained political commitment, innovative solutions for supply chain management and integrated malaria control programmes. Ultimately, adaptive, evidence-based interventions backed by ongoing research and regional cooperation are essential to achieving long-term malaria control and eventual eradication.

## Data Availability

Data are available upon reasonable request.

## References

[1] World malaria report 2024. https://www.who.int/teams/global-malaria-programme/reports/world-malaria-report-2024.

[2] Eastman RT, Fidock DA (2009). Artemisinin-based combination therapies: a vital tool in efforts to eliminate malaria. Nat Rev Microbiol.

[3] World Health Organization (2010). Global report on antimalarial drug efficacy and drug resistance: 2000-2010.

[4] Fanello CI, Karema C, van Doren W (2007). A randomised trial to assess the safety and efficacy of artemether-lumefantrine (Coartem) for the treatment of uncomplicated Plasmodium falciparum malaria in Rwanda. Trans R Soc Trop Med Hyg.

[5] Assefa A, Fola AA, Tasew G (2024). Emergence of Plasmodium falciparum strains with artemisinin partial resistance in East Africa and the Horn of Africa: is there a need to panic?. Malar J.

[6] Uwimana A, Umulisa N, Venkatesan M (2021). Association of Plasmodium falciparum kelch13 R561H genotypes with delayed parasite clearance in Rwanda: an open-label, single-arm, multicentre, therapeutic efficacy study. Lancet Infect Dis.

[7] Schreidah C, Giesbrecht D, Gashema P (2024). Expansion of artemisinin partial resistance mutations and lack of histidine rich protein-2 and -3 deletions in Plasmodium falciparum infections from Rukara, Rwanda. Malar J.

[8] Wernsman Young N, Gashema P, Giesbrecht D (2025). High Frequency of Artemisinin Partial Resistance Mutations in the Great Lakes Region Revealed Through Rapid Pooled Deep Sequencing. J Infect Dis.

[9] Kirby R, Giesbrecht D, Karema C (2023). Examining the Early Distribution of the Artemisinin-Resistant Plasmodium falciparum kelch13 R561H Mutation in Areas of Higher Transmission in Rwanda. Open Forum Infect Dis.

[10] Uwimana A, Legrand E, Stokes BH (2020). Emergence and clonal expansion of in vitro artemisinin-resistant Plasmodium falciparum kelch13 R561H mutant parasites in Rwanda. Nat Med.

[11] Multiple first-line therapies as part of the response to antimalarial drug resistance: an implementation guide.

[12] Zani B, Gathu M, Donegan S (2014). Dihydroartemisinin-piperaquine for treating uncomplicated Plasmodium falciparum malaria. Cochrane Database Syst Rev.

[13] Han KT, Lin K, Han ZY (2020). Efficacy and Safety of Pyronaridine-Artesunate for the Treatment of Uncomplicated Plasmodium falciparum and Plasmodium vivax Malaria in Myanmar. Am J Trop Med Hyg.

[14] Garcia-Vidal J, Ngirabega JD, Soldevila M (1989). Evolution of resistance of Plasmodium falciparum to antimalarial drugs in Rwanda, 1985-1987. Trans R Soc Trop Med Hyg.

[15] Gascón J, Soldevila M, Merlos A (1985). FALCIPARUM MALARIA IN RWANDA. The Lancet.

[16] Deloron P, Sexton JD, Bugilimfura L (1988). Amodiaquine and Sulfadoxine-Pyrimethamine as Treatment for Chloroquine-Resistant Plasmodium falciparum in Rwanda. Am J Trop Med Hyg.

[17] The East African Network for Monitoring Antimalarial Treatment (EANMAT) (2001). Monitoring antimalarial drug resistance within National Malaria Control Programmes: the EANMAT experience. Tropical Med Int Health.

[18] Rwagacondo CE, Niyitegeka F (2003). Efficacy of amodiaquine alone and combined with sulfadoxine-pyrimethamine and of sulfadoxine pyrimethamine combined with artesunate. Am J Trop Med Hyg.

[19] Rwagacondo CE, Karema C, Mugisha V (2004). Is amodiaquine failing in Rwanda? Efficacy of amodiaquine alone and combined with artesunate in children with uncomplicated malaria. Trop Med Int Health.

[20] Karema C, Fanello CI, van Overmeir C (2006). Safety and efficacy of dihydroartemisinin/piperaquine (Artekin) for the treatment of uncomplicated Plasmodium falciparum malaria in Rwandan children. Trans R Soc Trop Med Hyg.

[21] Zwang J, Ashley EA, Karema C (2009). Safety and efficacy of dihydroartemisinin-piperaquine in falciparum malaria: a prospective multi-centre individual patient data analysis. PLoS ONE.

[22] Zwang J, Olliaro P, Barennes H (2009). Efficacy of artesunate-amodiaquine for treating uncomplicated falciparum malaria in sub-Saharan Africa: a multi-centre analysis. Malar J.

[23] Sagara I, Rulisa S, Mbacham W (2009). Efficacy and safety of a fixed dose artesunate-sulphamethoxypyrazine-pyrimethamine compared to artemether-lumefantrine for the treatment of uncomplicated falciparum malaria across Africa: a randomized multi-centre trial. Malar J.

[24] Four Artemisinin-Based Combinations (4ABC) Study Group (2011). A head-to-head comparison of four artemisinin-based combinations for treating uncomplicated malaria in African children: a randomized trial. PLoS Med.

[25] Karema C, Imwong M, Fanello CI (2010). Molecular correlates of high-level antifolate resistance in Rwandan children with Plasmodium falciparum malaria. Antimicrob Agents Chemother.

[26] Lucchi N, Umulisa N, Harerimana JM (2022). Uncorrected Efficacy of artemether-lumefantrine for the treatment of uncomplicated Plasmodium falciparum infection in Rwanda.

[27] Subregional networks of antimalaria drug efficacy and resistance in eastern africa and horn of Africa countries. https://www.who.int/publications/i/item/9789240106758.

[28] Tumwebaze PK, Conrad MD, Okitwi M (2022). Decreased susceptibility of Plasmodium falciparum to both dihydroartemisinin and lumefantrine in northern Uganda. Nat Commun.

[29] Schallenberg E, van Loon W, Mbarushimana D (2025). Prevalence of Plasmodium falciparum Drug Resistance Markers pfcrt K76T and pfaat1 S258L in Southern Rwanda, 2010 to 2023. J Infect Dis.

[30] Straimer J, Gandhi P, Renner KC (2022). High Prevalence of Plasmodium falciparum K13 Mutations in Rwanda Is Associated With Slow Parasite Clearance After Treatment With Artemether-Lumefantrine. J Infect Dis.

[31] Bergmann C, van Loon W, Habarugira F (2021). Increase in Kelch 13 Polymorphisms in Plasmodium falciparum, Southern Rwanda. *Emerg Infect Dis*.

[32] Meier-Scherling CPG, Watson OJ, Asua V (2023). Selection of artemisinin partial resistance Kelch13 mutations in Uganda in 2016-22 was at a rate comparable to that seen previously in South-East Asia. medRxiv.

[33] Juliano JJ, Giesbrecht DJ, Simkin A Country wide surveillance reveals prevalent artemisinin partial resistance mutations with evidence for multiple origins and expansion of high level sulfadoxine-pyrimethamine resistance mutations in northwest tanzania. medRxiv.

[34] (2025). Rwanda_Malaria_NSP_Mid_Term_Report_2023.pdf. https://www.rbc.gov.rw/fileadmin/user_upload/report_2024/Rwanda_Malaria_NSP_Mid_Term_Report_2023.pdf.

[35] (2025). MOPDD_Annual_Report_FY2023-2024.pdf. https://rbc.gov.rw/fileadmin/user_upload/report_2024/malaria_2025/MOPDD_Annual_Report_FY2023-2024.pdf.

[36] (2025). RBC: RBC and Zipline introduce drone delivery of Malaria medicines. https://rbc.gov.rw/media/details?tx_news_pi1%5Baction%5D=detail&tx_news_pi1%5Bcontroller%5D=News&tx_news_pi1%5Bnews%5D=1113&cHash=af33f97230085d91c6a3bbe5b1cdfbc4.

[37] Talisuna AO, Karema C, Ogutu B (2012). Mitigating the threat of artemisinin resistance in Africa: improvement of drug-resistance surveillance and response systems. Lancet Infect Dis.

[38] (2022). Strategy to respond to antimalarial drug resistance in Africa.

[39] Lertpiriyasuwat C, Sudathip P, Kitchakarn S (2021). Implementation and success factors from Thailand’s 1-3-7 surveillance strategy for malaria elimination. Malar J.

[40] Novotny J, Singh A, ACTwatch Group (2016). Evidence of successful malaria case management policy implementation in Cambodia: results from national ACTwatch outlet surveys. Malar J.

[41] Sagara I, Rulisa S, Mbacham W (2009). Efficacy and safety of a fixed dose artesunate-sulphamethoxypyrazine-pyrimethamine compared to artemether-lumefantrine for the treatment of uncomplicated falciparum malaria across Africa: a randomized multi-centre trial. Malar J.

[42] The Four Artemisinin-Based Combinations (4ABC) Study Group A Head-to-Head Comparison of Four Artemisinin-Based Combinations for Treating Uncomplicated Malaria in African Children: A Randomized Trial. PLoS Med.

[43] Uwimana A, Penkunas MJ, Nisingizwe MP (2019). Efficacy of artemether-lumefantrine versus dihydroartemisinin-piperaquine for the treatment of uncomplicated malaria among children in Rwanda: an open-label, randomized controlled trial. Trans R Soc Trop Med Hyg.

